# Prescrição de Exercício Aeróbio na Reabilitação Cardíaca Baseada na Frequência Cardíaca dos Estágios do Teste da Fala e do Teste de Caminhada de 6 Minutos

**DOI:** 10.36660/abc.20230086

**Published:** 2023-09-19

**Authors:** Amanda Althoff, Ariany Marques Vieira, Lucas Santos da Silveira, Magnus Benetti, Marlus Karsten

**Affiliations:** 1 PPGFT Universidade do Estado de Santa Catarina Florianópolis SC Brasil Programa de Pós-Graduação em Fisioterapia (PPGFT) – Universidade do Estado de Santa Catarina (UDESC), Florianópolis, SC – Brasil; 2 GEPCardio Universidade do Estado de Santa Catarina Florianópolis SC Brasil Grupo de Pesquisa em Saúde Cardiovascular e Exercício (GEPCardio) – Universidade do Estado de Santa Catarina (UDESC), Florianópolis, SC – Brasil; 3 Montreal Behavioral Medicine Centre CIUSSS du Nord-de-l’Île-de-Montréal Montreal Canadá Montreal Behavioral Medicine Centre, CIUSSS du Nord-de-l’Île-de-Montréal, Montreal – Canadá; 4 PPGCMH Universidade do Estado de Santa Catarina Florianópolis SC Brasil Programa de Pós-Graduação em Ciências do Movimento Humano (PPGCMH) – Universidade do Estado de Santa Catarina (UDESC), Florianópolis, SC – Brasil

**Keywords:** Reabilitação Cardiovascular, Exercício Físico, Teste da Fala

## Abstract

**Fundamento:**

Embora o Teste da Fala (TF) seja um teste confiável e de baixo custo, seu uso para prescrição de exercício aeróbio ainda é limitado.

**Objetivo:**

Analisar a frequência cardíaca (FC) dos estágios do TF e no pico do teste de caminhada de 6 minutos (TC6min) como parâmetro para a prescrição de exercício aeróbio comparando com a FC no primeiro e segundo limiares ventilatórios (LV1 e LV2) do teste cardiopulmonar de exercício (TCPE).

**Métodos:**

Pacientes com doença cardiovascular compareceram a 3 dias de avaliação: 1) anamnese e TCPE; 2) TC6min; e 3) TF. Foram usados ANOVA unidirecional de medidas repetidas ou teste de Friedman para comparar a FC no LV1 e LV2 com a FC nos estágios do TF: último positivo (TF+), primeiro equívoco (TF±) e negativo (TF−) e no pico do TC6min. O teste de Pearson ou Spearman avaliou se há correlação entre FC em LVs, estágios do TF e TC6min. A significância estatística foi fixada em 5%.

**Resultados:**

O estudo incluiu 22 pacientes cardíacos (13 homens, 61 ± 8 anos). A FC no LV1 foi semelhante à FC no TF+ (p = 0,987) e TF± (p = 0,154), e moderadamente correlacionada com o TF+ (r = 0,479, p = 0,024). A FC no LV2 foi semelhante ao TF− (p = 0,383), com forte correlação (r = 0,757, p < 0,001). A FC no pico do TC6min foi significativamente diferente da FC no TF+, TF± e LV1 (p = 0,001, p = 0,005 e p < 0,001, respectivamente), mas semelhante ao TF− (p = 0,68).

**Conclusões:**

A FC no TF+ e TF− reflete a FC no LV1 e LV2, respectivamente, diferentemente do TC6min, que foi semelhante apenas ao LV2. O TF pode ser um teste objetivo para auxiliar a prescrição de exercício aeróbio na reabilitação cardíaca.

## Figura Central

**Figure:**
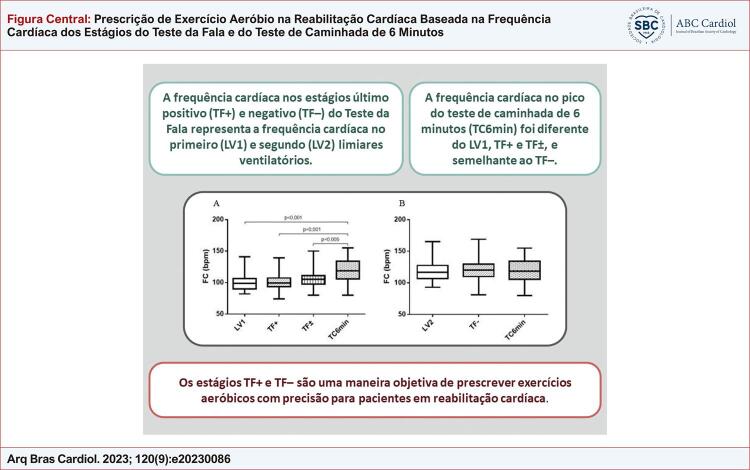
: Prescrição de Exercício Aeróbio na Reabilitação Cardíaca Baseada na Frequência Cardíaca dos Estágios do Teste da Fala e do Teste de Caminhada de 6 Minutos

## Introdução

O principal objetivo da reabilitação cardiovascular é melhorar a aptidão cardiorrespiratória por meio do exercício aeróbio (EA).^1^ A recomendação geral para a prática de exercício físico, segundo a Sociedade Brasileira de Cardiologia, é de 150 minutos semanais divididos em 3 a 5 sessões semanais. A prática adequada de exercício físico visa reduzir a pressão arterial, o risco de eventos cardiovasculares e o risco de mortalidade em pacientes cardiovasculares.^1,2^

O teste cardiopulmonar de exercício (TCPE) é o teste de referência para avaliar a função cardiorrespiratória e prescrever a intensidade do EA.^1^ A análise do TCPE permite a identificação das variáveis mais adequadas e recomendadas, como consumo máximo de oxigênio, carga de trabalho, frequência cardíaca (FC) máxima, FC de reserva e limiares ventilatórios (LVs).^3-5^ No entanto, esse teste ainda tem acesso limitado, principalmente em países em desenvolvimento, devido aos altos custos, à necessidade de profissionais altamente capacitados e às análises complexas.^4,5^

Nesse sentido, métodos alternativos ao TCPE têm sido utilizados, como equações para predizer a FC máxima e escalas relacionadas à percepção subjetiva de esforço.^6^ Outros métodos utilizam valores de FC absolutos ou relativos obtidos durante testes submáximos, como o teste de caminhada de 6 minutos (TC6min)^7-9^ e o teste da fala (TF).^10,11^ O TF é um teste validado e acessível, baseado em um protocolo de carga incremental e utiliza a percepção do conforto de fala como marcador da intensidade do exercício.^12,13^ Recentemente, o TF tem sido recomendado para avaliar a capacidade funcional e prescrever EA por diretrizes nacionais e internacionais de prevenção e reabilitação cardiovascular.^1,2^

O TF pode ser aplicado em cicloergômetro ou esteira, comumente de forma semelhante ao protocolo do TCPE. Ao final de cada estágio, o paciente tem seu conforto de fala questionado, geralmente lendo um parágrafo padrão e respondendo à pergunta “Está confortável para falar?”. Existem 3 alternativas de resposta: “sim”, a fala continua confortável durante o exercício (TF positivo, TF+); “talvez”, o paciente percebe desconforto na fala (TF incerto ou equívoco, TF±); e “não”, o paciente não consegue ler o parágrafo confortavelmente (TF negativo, TF−). O último estágio (TF−) é considerado um critério para interrupção do teste.^12,14-16^ No entanto, estudos utilizando TF para prescrever EA são escassos, mesmo em pessoas saudáveis.

Não se sabe se a FC alcançada nos estágios do TF é uma medida válida para prescrever a intensidade do EA e se esses estágios podem ser comparados com a FC no pico do TC6min e nos LVs do TCPE. Acreditamos que a FC nos estágios do TF esteja associada ao padrão de referência, aos valores dos LVs, bem como com a frequência cardíaca pico (FCpico) alcançada em um TC6min. Compreender as semelhanças e as associações entre essas 3 diferentes ferramentas de prescrição pode facilitar a prática clínica, fornecendo informações sobre uma ferramenta adicional para prescrever EA, que pode fornecer um parâmetro de intensidade independente, individualizado e confiável. Portanto, objetivamos analisar e comparar a FC nos estágios do TF e na FCpico no TC6min, bem como compará-las com a FC nos LVs do TCPE.

## Métodos

O presente estudo foi aprovado pelo Comitê de Ética em Pesquisa com Seres Humanos da Universidade do Estado de Santa Catarina (número 96032818.4.0000.0118) e conduzido de acordo com a Resolução 466/12 do Conselho Nacional de Saúde e a Declaração de Helsinque. Todos os pacientes assinaram o termo de consentimento livre e esclarecido.

### Participantes

Uma amostra intencional e não probabilística de pacientes de ambos os sexos, com idade entre 40 e 80 anos, diagnosticados com doença cardiovascular crônica clinicamente estável (sem história de internação hospitalar ou mudança de medicação nas 4 semanas anteriores ao estudo) foi recrutada no Núcleo de Cardio-oncologia e Medicina do Exercício da Universidade do Estado de Santa Catarina.

Os critérios de inclusão foram alfabetização para compreender suficientemente o TF e assinar o termo de consentimento livre e esclarecido. Os critérios de exclusão foram alterações de fala ou musculoesqueléticas, acuidade visual comprometida que impedisse o paciente de ler o parágrafo, dor intensa inespecífica ou angina durante os testes, doença neurológica diagnosticada por meio de teste cognitivo ou doenças respiratórias.

### Coleta de dados

Os dados foram coletados em 3 dias. O primeiro dia consistiu em uma entrevista estruturada e medidas antropométricas, incluindo índice de massa corporal, avaliação cognitiva (mini exame do estado mental) e avaliação do TCPE. No segundo dia, foram realizados dois TC6min com intervalo de 30 minutos entre eles. O TF foi realizado no terceiro dia. Todos os pacientes foram avaliados dentro de 48 e 72 horas entre os testes e no mesmo período do dia.

### Mini exame do estado mental

O mini exame do estado mental foi utilizado como critério de inclusão. Este exame foi aplicado com uma pontuação de 0 a 30 com pontos de corte de acordo com o nível de educação: analfabeto (20 pontos), 1 a 4 anos de estudo (25 pontos), 5 a 8 anos de estudo (26,5 pontos), 9 a 11 anos de estudo (28 pontos) e > 11 anos de estudo (29 pontos).^17^

### Teste cardiopulmonar de exercício

O TCPE foi realizado em esteira (ATL, Inbramed, Porto Alegre, RS, Brasil) utilizando um protocolo de rampa individualizado. A velocidade e a inclinação variaram a cada minuto para permitir que o teste durasse entre 8 e 12 minutos. Os pacientes realizaram recuperação ativa após o protocolo (1 minuto usando a velocidade inicial).

Utilizamos um circuito aberto computadorizado (Quark CPET, COSMED, Itália) para analisar os gases expiratórios e a ventilação, enquanto um frequencímetro de pulso (Polar® RS800 CX, Kempele, Finlândia) avaliou a FC durante o teste. O primeiro (LV1) e o segundo (LV2) LV foram identificados usando os métodos V-slope e PetO_2_-PetCO_2_ (Quark CPET, COSMED, Itália).

Para a segurança dos pacientes, foram adotados os seguintes critérios de interrupção do teste: sinais ou sintomas de intolerância ao exercício (dor ou desconforto torácico, dispneia exacerbada, tontura ou confusão, ataxia, palidez, sudorese excessiva, cianose, claudicação ou cãibras), resposta cardiovascular inadequada (pressão arterial e/ou FC), ou solicitação de interrupção pelo participante.

### Teste de caminhada de 6 minutos

O TC6min foi aplicado de acordo com as diretrizes atuais.^18^ Duas repetições do TC6min foram realizadas em uma pista de 30 metros de extensão, e os pacientes foram solicitados a caminhar a maior distância possível em 6 minutos.^18^

A FC (Polar® RS800 CX, Kempele, Finlândia), a pressão arterial (Aneroid Calibra®, MDF Instruments, Porto Rico, EUA), a saturação de pulso de oxigênio (AT101C, Bioland, Taiwan) e o esforço percebido (escala Borg CR10)^19^ foram avaliados antes, imediatamente após e 2 minutos após os testes. A FC e a saturação de pulso de oxigênio também foram avaliadas a cada minuto durante os testes.^18^ Para análise, utilizamos a FCpico apresentada durante o teste com a maior distância percorrida. Os critérios para interrupção do TC6min foram semelhantes aos do TCPE.

### Teste da fala

O TF foi realizado em esteira (Embreex 570 Pro, Brusque, SC, Brasil) usando um protocolo incremental independente e individualizado de acordo com a distância predita do TC6min. A FC, pressão arterial, saturação de pulso de oxigênio e esforço percebido foram avaliados antes, imediatamente após e 2 minutos após o TF. Também monitoramos a FC e a saturação de pulso de oxigênio durante o teste, enquanto o esforço percebido foi questionado apenas ao final de cada estágio.

A carga foi aumentada a cada 2 minutos durante o teste (estágios). Nos últimos 30 segundos de cada estágio, solicitamos aos pacientes que recitassem o seguinte parágrafo de 36 palavras:

“Saúde é um estado de completo bem-estar físico, mental e social, e não consiste apenas na ausência de doença ou de enfermidade. É um direito fundamental, que deve ser assegurado sem distinção de raça, religião ou condição social.”

Logo após a leitura, foi perguntado aos pacientes “Está confortável para falar?”, e as respostas possíveis eram “sim” (TF+); “mais ou menos” (TF±); ou “não” (TF−). O TF− foi considerado um critério para interromper o TF. Outros critérios para interrupção do teste foram semelhantes ao TCPE e TC6min.

### Protocolo do TF baseado no TC6min

Um protocolo incremental contínuo foi conduzido com incrementos de velocidade ou inclinação no início de cada estágio. Foi usada uma equação para calcular a distância predita do TC6min (TC6minpred).^20^


$$\begin{array}{cc} & \text{ TC6minpred }=890,46-(6,11\times \text{ idade })+\left(0,0345\times {\text{ idade }}^{2}\right)\\ & \;+(48,87\times \text{ sexo })-(4.87\times \text{ índice de massa corporal }) \end{array}$$

**Figura 1:**
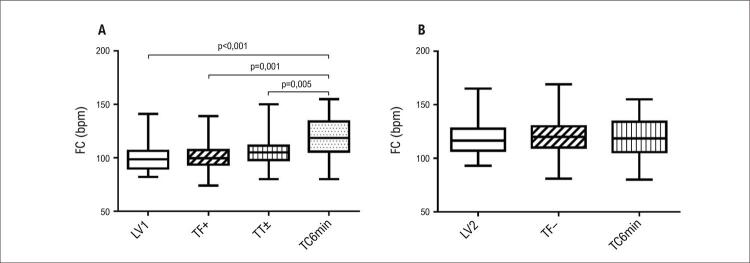
– A: Comparação da FC entre LV1, estágiosTF+ e TF± e TC6min. B: Comparação da FC entre LV2, TF- e TC6min. Fonte: elaborada pelos autores. Nota: devido à distribuição não normal e normal, foi usado o teste de Friedman para as variáveis da Figura 1A e ANOVA de medidas repetidas para as variáveis da Figura 1B, respectivamente.

Conhecendo o TC6minpred, a velocidade média foi estimada da forma seguinte:  TFvelocidade (km/h)=( TC6minpred [m]×10[min])/1000 ; equivalente a 100% da velocidade média estimada durante o TC6min. Também calculamos os percentuais correspondentes a cada estágio do TF, iniciando em 70% e aumentando 10 pontos percentuais a cada 2 minutos até 110% da TFvelocidade. A inclinação da esteira foi mantida em 2% até o primeiro estágio em 110% da velocidade. A partir daí, foram aumentados 2 pontos percentuais a cada estágio até o final do protocolo (Figura 1S, Material Suplementar).

### Análise estatística

Os dados foram analisados usando o software Statistical Package for the Social Sciences, versão 20.0 (SPSS, IBM Corporation, Armonk, EUA). As variáveis contínuas foram descritas como média e desvio padrão ou mediana e intervalo interquartílico, de acordo com a normalidade dos dados. A normalidade da distribuição dos dados foi verificada com o teste de Shapiro-Wilk.

As variáveis com distribuição normal foram comparadas por meio da ANOVA de medidas repetidas, seguida do post-hoc de Bonferroni, enquanto o teste de Friedman foi usado para variáveis com distribuição não normal. O coeficiente de correlação de Pearson ou Spearman avaliou a associação entre as variáveis. O nível de significância adotado na análise estatística foi de 5%.

### Cálculo do tamanho da amostra

O tamanho da amostra foi calculado usando o GPower 3.1 em um estudo piloto com 5 participantes, considerando nível de significância bidirecional (α) de 5% e poder de 80% (β = 0,20). Com base na correlação entre FC durante os estágios do TF (TF+ e TF±) e LV1 (TF+: r = 0,95; TF±: r = 0,96) e FCpico no TC6min (TF+: r = 0,56; TF±: r = 0,57), o tamanho amostral mínimo estimado foi de 20 pacientes.

## Resultados

Avaliamos 30 pacientes, dos quais 8 foram excluídos devido a alterações musculoesqueléticas (n = 3), erro de medida (n = 3), angina (n = 1) e dor periumbilical (n = 1). Dos 22 pacientes incluídos, 13 eram do sexo masculino (59,1%) (Tabela 1).

**Tabela 1:** – Características descritivas dos sujeitos

Variável	Média (DP)	n	%
**Idade (anos)**	61,1 (8,5)		
**Índice de massa corporal (kg/m^2^)**	29,5 (4,1)		
**FC (bpm)**			
Repouso (antes do TCPE)	73,6 (1,8)		
Repouso (antes do TC6min)	74,4 (2,2)		
Repouso (antes do TF)	74,1 (1,9)		
LV1	100,9 (14,8)		
LV2	119,0 (16,1)		
TC6min	119,4 (20,1)		
TF+	101,1 (14,7)		
TF±	105,4 (15,7)		
TF−	121,4 (18,4)		
**Distância prevista no TC6min (m)**	530,7 (42,9)		
**Distância real no TC6min (m)**	577,8 (87,9)		
**Distância real em relação à prevista no TC6min (%)**	108,9 (14,9)		
**Diagnóstico cardíaco**			
Doença arterial coronariana		19	86,4
Insuficiência cardíaca		1	4,5
Doença arterial coronariana + insuficiência cardíaca		9	40,9
Cardiomiopatia hipertrófica		1	4,5
Doenças das válvulas cardíacas		2	9,1
**Comorbidades**			
Obesidade		9	40,9
Dislipidemia		15	68,2
Hipertensão		19	86,4
Diabetes mellitus		9	40,9
Ex-fumante		9	40,9
**Angioplastia**		13	59,1
**Cirurgia cardíaca**			
Revascularização miocárdica		10	45,5
Substituição da válvula		2	9,1
**Tempo no programa de reabilitação (meses)**			
1–6		11	50,0
6–12		1	4,5
12–24		4	18,2
≥24		6	27,3

DP: desvio padrão; FC: frequência cardíaca; LV1: primeiro limiar ventilatório; LV2: segundo limiar ventilatório; TC6min: teste de caminhada de 6 minutos; TCPE: teste cardiopulmonar de exercício; TF: teste da fala; TF+: último estágio positivo da TF; TF±: primeiro estágio equívoco do TF. Fonte: elaborada pelos autores.

Conforme demonstrado na Tabela 1, não foi encontrada diferença significativa entre a FC de repouso antes do TCPE, TF e TC6min (p = 1,0).

A Figura 1 apresenta a comparação entre a FC nos LVs, TF+, TF±, TF− e FCpico no TC6min. Encontramos uma diferença significativa entre a FCpico no TC6min e a FC no LV1, TF+ e TF± (p < 0,001, p = 0,001 e p = 0,005, respectivamente). Não foi observada diferença entre a FC no LV1 e TF+ (p = 0,987) e TF± (p = 0,154) (Figura 1A) ou entre LV2 e TF− (p = 0,383). A FCpico no TC6min foi semelhante à FC no TF− (p = 0,68) e LV2 (p = 0,92) (Figura 1B).

Uma correlação moderada foi verificada entre TF+ e LV1. No entanto, a FC no TF± não se correlacionou com a FC no LV e a FCpico no TC6min. A FC no TF− foi fortemente correlacionada com o LV2. A FCpico no TC6min não foi correlacionada com a FC no LV1, mas sim com a FC no VT2 e TF−. A Tabela 2 apresenta todas as correlações da FC nos estágios do TF, LV e FCpico no TC6min.

**Tabela 2:** – Correlações da FC nos estágios do TF, LV1 e FCpico no TC6min

	FC no LV1		FCpico (TC6min)	

	rho	p	r	p
TF+	0,479	0,024*	0,384	0,078
TF±	0,412	0,057	0,357	0,103

LV1: primeiro limiar ventilatório; TC6min: teste de caminhada de 6 minutos; TF: teste da fala; TF+: último estágio positivo da TF; TF±: primeiro estágio equívoco do TF. *p < 0,05. Fonte: elaborada pelos autores.

## Discussão

Este é o primeiro estudo que compara a FC avaliada nos LVs do TCPE com a FC nos estágios de um protocolo de TF independente e individualizado e durante um teste de campo (TC6min). A FC no TF+ e TF± foi semelhante à FC no LV1, enquanto a FC no TF− e a FCpico no TC6min foram semelhantes à FC no LV2. A FC no TF+ e TF− se correlacionou com a FC no VT1 e VT2, respectivamente. Não foi encontrada correlação entre a FCpico no TC6min e a FC nos estágios do TF.

Os métodos individualizados de prescrição de EA mais reconhecidos são o consumo máximo de oxigênio e os LVs identificados pelo TCPE.^1,4^ Se um TCPE não estiver disponível, o teste ergométrico pode ser usado para obter parâmetros cardiovasculares máximos. Na ausência desses testes, a avaliação funcional e a prescrição de EA geralmente são realizadas com base em um teste de campo, como o TC6min e o TF.^8^ Embora os estágios do TF estejam fisiologicamente relacionadas aos LVs, seu uso para prescrição de EA ainda é escasso, principalmente para pacientes cardíacos.

No presente estudo, investigamos um protocolo de TF e encontramos correlações entre a FC no LV1 e TF+ e TF±. O TF não possui um protocolo padronizado e pode ser realizado com diferentes equipamentos, progressões de carga e durações dos estágios, e a fala pode ser desafiada usando parágrafos ou contagem.^16^ Apesar de utilizarem métodos diferentes, estudos demonstraram semelhanças entre a FC e o consumo de oxigênio nos estágios do TF e LV em pacientes com doenças cardíacas, sugerindo que os estágios do TF podem ser usadas para prescrever EA.^14,15,21,22^

Nossos resultados foram semelhantes aos de Brawer et al. (2006), que aplicaram o TF em pacientes com doença arterial coronariana estável e demonstraram nenhuma diferença na estimativa do LV1 e na viabilidade da prescrição de EA pela FC de acordo com o TF.^22^ Além disso, foi encontrada uma forte correlação entre a FC no TF− e LV2. Embora haja escassez de estudos comparando a FC entre esses dois momentos em pacientes com doença cardiovascular, associações entre o consumo de oxigênio e a FC no TF− e LV2 foram encontradas em indivíduos saudáveis e atletas,^23,24^ sugerindo, portanto, que o TF− pode orientar a prescrição de EA e refletir a prescrição com base no LV2.

Outro método comumente utilizado em programas de reabilitação cardiovascular para prescrição da intensidade de EA é baseado no TC6min. Gremeaux et al. (2011) compararam os efeitos de 3 prescrições individualizadas de treinamento físico em indivíduos treinando em intensidade moderada. Encontraram valores semelhantes entre a FCpico no TC6min e a FC alvo recomendada. Outro estudo de Calegari et al. compararam a FC no LV1 durante os últimos 30 segundos do TC6min em pacientes com doença arterial coronariana em tratamento com betabloqueadores. Os autores encontraram concordância entre a FC avaliada ao final do TC6min e o LV1, sugerindo que a FC ao final do TC6min foi adequada para prescrever e monitorar o EA nessa população.^25^

Esses achados corroboram a ideia de utilizar a FC no TC6min como método de prescrição da intensidade do exercício, a FC medida no TC6min sendo semelhante à FC avaliada no LV1. No entanto, encontramos diferenças entre a FCpico no TC6min e a FC no LV1 em nosso estudo, enquanto a FCpico no TC6min foi semelhante à FC no LV2 e no TF−. De acordo com as recomendações mais recentes, a prescrição de EA deve ser realizada entre o primeiro e o segundo VT;^1^ nossos dados sugerem que a FCpico no TC6min pode não ser adequada para prescrever o limite inferior de intensidade de EA em pacientes com doença cardiovascular crônica. Além disso, a FCpico no TC6min, como LV2 e TF−, pode representar o limite superior da prescrição de EA.

Essa semelhança nunca foi descrita na literatura e alguns aspectos do presente estudo podem ter influenciado esse resultado. Os pacientes faziam parte de um programa de reabilitação cardiovascular que realiza regularmente o TC6min. Além disso, em relação à distância percorrida, nossa amostra atingiu uma distância percorrida acima de 100% do previsto, demonstrando pouco comprometimento da capacidade funcional. Além disso, o TC6min foi realizado em campo aberto, enquanto o TCPE e o TF foram realizados em ambientes controlados. Adicionalmente, estudos anteriores utilizaram a FC do minuto final do TC6min em vez da FCpico durante o teste, que foi utilizada em nossas análises.

Embora comumente utilizado na reabilitação cardiovascular, o TC6min requer espaço físico devido à pista de 30 metros de extensão. Além disso, o teste é auto-cadenciado e apresenta variações inter e intraindividuais de velocidade e desempenho.^18^ Por outro lado, o TF requer menos espaço físico, pode ser realizado em cicloergômetro ou esteira, segue um protocolo de rampa incremental semelhante ao TCPE, e é de fácil aplicação e equivalente ao padrão de referência.

O presente estudo não está isento de limitações. Os pacientes faziam parte de um programa de reabilitação cardiovascular fase III e possivelmente apresentavam melhores condições físicas do que os pacientes nas fases iniciais da reabilitação. Além disso, a percepção da fala confortável é subjetiva e foram perdidos alguns dados devido a erro de mensuração.

As comparações entre o TF e o TCPE foram semelhantes à literatura, exceto entre o TF e TC6min. Acreditamos que o TF apresente parâmetros associados a variáveis fisiológicas e do TCPE (por exemplo, LVs). Nossos resultados também mostraram a aplicabilidade clínica do TF como uma ferramenta fácil, segura e individualizada para a prescrição de EA.

## Conclusão

De acordo com o protocolo proposto, a FC no TF+ e TF− reflete a FC no LV1 e LV2, respectivamente, demonstrando que o TF é um teste objetivo e de baixo custo para auxiliar a prescrição de EA em pacientes com doenças cardiovasculares crônicas. Por outro lado, a prescrição de EA utilizando a FCpico no TC6min deve ser realizada com cautela, pois alguns estudos mostraram sua correlação com o LV1, diferentemente do que foi verificado em nosso estudo, no qual a FCpico no TC6min foi semelhante à FC no LV2 e TF−. Sugerimos futuros ensaios clínicos randomizados usando parâmetros do TF para prescrever EA em pacientes com doenças cardiovasculares.
