# Supplementary material for: Prescrição de Exercício Aeróbio na Reabilitação Cardíaca Baseada na Frequência Cardíaca dos Estágios do Teste da Fala e do Teste de Caminhada de 6 Minutos
Source: Arq Bras Cardiol. 2023 Sep 19;120(9):e20230086. [Article in Portuguese] doi: 10.36660/abc.20230086 (PMC10519355; doi:10.36660/abc.20230086)
Supplement: Translation

# Translation: Aerobic Exercise Prescription in Cardiac Rehabilitation Based on Heart Rate from Talk Test Stages and 6-Minute Walk Test

**Authors:** Amanda Althoff, Ariany Marques Vieira, Lucas Santos da Silveira, Magnus Benetti, Marlus Karsten

**Affiliations:** 1 Brasil Programa de Pós-Graduação em Fisioterapia (PPGFT) – Universidade do Estado de Santa Catarina (UDESC), Florianópolis, SC – Brasil; 2 Brasil Grupo de Pesquisa em Saúde Cardiovascular e Exercício (GEPCardio) – Universidade do Estado de Santa Catarina (UDESC), Florianópolis, SC – Brasil; 3 Canadá Montreal Behavioral Medicine Centre, CIUSSS du Nord-de-l’Île-de-Montréal, Montreal – Canadá; 4 Brasil Programa de Pós-Graduação em Ciências do Movimento Humano (PPGCMH) – Universidade do Estado de Santa Catarina (UDESC), Florianópolis, SC – Brasil

**Keywords:** Cardiac Rehabilitation, Exercise, Talk Test

## Abstract

**Background:**

Although the Talk Test (TT) is a reliable and low-cost test, its use for aerobic exercise prescription is still limited.

**Objective:**

To analyze the heart rate (HR) in the stages of the TT and at the peak of the 6-minute walk test (6MWT) as a parameter to prescribe aerobic exercise compared with HR at the first and second ventilatory thresholds (VT1 and VT2) of cardiopulmonary exercise test (CPET).

**Methods:**

Individuals with cardiovascular disease attended three assessment days: 1) anamnesis and CPET; 2) 6MWT; and 3) TT. One-way repeated measures ANOVA or Friedman’s test were used to compare HR at VT1 and VT2 with HR at TT stages: last positive (TT+), first equivocal (TT±), and negative (TT−), and at the peak of the 6MWT. Pearson’s or Spearman’s test assessed correlations between HR at VTs, TT stages, and 6MWT. Statistical significance was set at 5%.

**Results:**

The study included 22 cardiac patients (13 men, 61 ± 8 years). HR at VT1 was similar to HR at TT+ (p = 0.987) and TT± (p = 0.154), and moderately correlated with TT+ (r = 0.479, p = 0.024). HR at VT2 was similar to TT− (p = 0.383), with a strong correlation (r = 0.757, p < 0.001). HR at the peak of the 6MWT was significantly different from HR at TT+, TT±, and VT1 (p = 0.001, p = 0.005, and p < 0.001, respectively) but similar to TT− (p = 0.68).

**Conclusions:**

HR at TT+ and TT− reflect HR at VT1 and VT2, respectively, differently from 6MWT, which was similar only to VT2. TT may be an objective test to assist aerobic exercise prescription in cardiac rehabilitation.

## Introduction

The main goal of cardiovascular rehabilitation is to improve cardiorespiratory fitness using aerobic exercise (AE).^1^ The general recommendation for the practice of physical exercise, according to the Brazilian Society of Cardiology, is 150 minutes per week divided into 3 to 5 weekly sessions. The appropriate practice of physical exercise aims to reduce blood pressure, risk of cardiovascular events and risk of mortality in cardiovascular patients.^1,2^

The cardiopulmonary exercise test (CPET) is the reference test to assess cardiorespiratory function and prescribe AE intensity.^1^ CPET analysis allows identification of the most adequate and recommended variables, such as maximal oxygen uptake, workload, maximal heart rate (HR), HR reserve, and ventilatory thresholds (VTs).^3-5^ However, this test still has limited access, especially in developing countries, due to high costs, the need for highly trained professionals, and complex analyses.^4,5^

In this sense, alternative methods to CPET have been used, such as equations to predict the maximal HR and scales related to subjective perceived exertion.^6^ Another method uses absolute or relative HR values achieved during submaximal tests, such as the 6-minute walk test (6MWT)^7-9^ and the talk test (TT).^10,11^ The TT is a validated and accessible test, based on an incremental load protocol, and it uses the perception of speech comfort as a marker for exercise intensity.^12,13^ Recently, the TT has been recommended to assess functional capacity and prescribe AE by national and international cardiovascular prevention and rehabilitation guidelines.^1,2^

The TT can be applied using a cycle ergometer or treadmill, commonly in a similar way to the CPET protocol. At the end of each stage, patients have their speech comfort challenged, usually reading a standard paragraph, and answering the question “Can you speak comfortably?”. There are 3 answer alternatives: “yes,” the speech is still comfortable during exercise (TT positive, TT+); “maybe,” the patient notices discomfort in the speech (TT uncertain or equivocal, TT±); and “no,” the patient is unable to read the paragraph comfortably (TT negative, TT−). The last stage (TT−) is considered a criterion for interrupting the test.^12,14-16^ Nevertheless, studies using TT to prescribe AE are scarce, even in healthy people.

It is unknown whether HR achieved at TT stages is a valid measure to prescribe the AE intensity and whether these stages can be compared with the HR at the peak of the 6MWT and at the VTs from a CPET. We believe that the HR at the TT stages is associated with the reference standard, the values on the VTs, as well as with the peak heart rate (HRpeak) achieved in a 6MWT. Understanding the similarities and associations between these 3 different prescription tools can facilitate clinical practice by providing information about an additional tool to prescribe AE, which can provide an independent, individualized, and reliable intensity parameter. Therefore, we aimed to analyze and compare the HR at the TT stages and at the HRpeak in the 6MWT, as well as to compare them with HR at VTs of CPET.

## Methods

This study was approved by the Human Research Ethics Committee (number 96032818.4.0000.0118) and conducted according to Resolution 466/12 of the National Health Council and the Declaration of Helsinki. All patients signed the informed consent form.

### Participants

An intentional and non-probability sample of patients of both sexes, aged between 40 and 80 years, diagnosed with clinically stable chronic cardiovascular disease (i.e., no history of hospitalization or medication change within 4 weeks before the study) was recruited in the Center of Cardio-Oncology and Exercise Medicine of Santa Catarina State University

Inclusion criteria were sufficient literacy to understand the TT and sign the informed consent form. Exclusion criteria were speech or musculoskeletal alterations, impaired visual acuity that prevented the patient from reading the paragraph, non-specific intense pain or angina during the tests, neurological disease diagnosed using a cognitive test, or respiratory diseases.

### Data collection

Data were collected in 3 days. The first day consisted of a structured interview and anthropometric measurements, including body mass index, cognitive assessment (mini-mental state exam), and CPET assessment. On the second day, two 6MWT were performed with a 30-minute interval in between. The TT was performed on the third day. All patients were evaluated within 48 and 72 hours between the tests at the same period of the day.

### Mini-mental state exam

The mini-mental state exam was used as an inclusion criterion. This exam was applied using a score from 0 to 30 with cut-off points according to educational level as follows: illiterate (20 points), 1 to 4 years of schooling (25 points), 5 to 8 years of schooling (26.5 points), 9 to 11 years of schooling (28 points), and > 11 years of schooling (29 points).^17^

### Cardiopulmonary exercise test

The CPET was performed on a treadmill (ATL, Inbramed, Porto Alegre, RS, Brazil) using an individualized ramp protocol. Speed and inclination varied each minute to enable the test to last between 8 and 12 minutes. Patients performed an active recovery after the protocol (1 minute using the initial speed).

We used a computerized open circuit (Quark CPET, COSMED, Italy) to analyze expiratory gases and ventilation, while a pulse rate monitor (Polar® RS800 CX, Kempele, Finland) assessed HR during the test. The first (VT1) and second (VT2) VT were identified using V-slope and PetO_2_-PetCO_2_ methods (Quark CPET, COSMED, Italy).

The following criteria for interrupting the test were adopted for the safety of patients: signs or symptoms of exercise intolerance (chest pain or discomfort, exacerbated dyspnea, dizziness or confusion, ataxia, pallor, excessive sweating, cyanosis, claudication, or cramps), inadequate cardiovascular response (blood pressure and/or HR), or if requested by the participant.

### 6-minute walk test

The 6MWT was applied according to current guidelines.^18^ Two repetitions of the 6MWT were performed on a 30-meter-long track, and patients were requested to walk the longest distance possible in 6 minutes.^18^

HR (Polar® RS800 CX, Kempele, Finland), blood pressure (Aneroid Calibra®, MDF Instruments, Puerto Rico, USA), pulse oxygen saturation (AT101C, Bioland, Taiwan), and perceived exertion (Borg CR10 scale)^19^ were assessed before, immediately after, and 2 minutes after the tests. HR and pulse oxygen saturation were also assessed each minute during the tests.^18^ For analysis we used the HRpeak presented during the test with the higher walked distance. Criteria for interrupting the 6MWT were similar to CPET.

### Talk test

The TT was performed on a treadmill (Embreex 570 Pro, Brusque, SC, Brazil) using an independent and individualized incremental protocol according to the predicted 6MWT distance. HR, blood pressure, pulse oxygen saturation, and perceived exertion were assessed before, immediately after, and 2 minutes after the TT. We also monitored HR and pulse oxygen saturation during the test, while perceived exertion was questioned only at the end of each stage.

The load was increased every 2 minutes during the test (stages). In the last 30 seconds of each stage, we requested patients to recite the following paragraph of 36 words:

“Health is a state of complete physical, mental, and social well-being, not only the absence of disease or illness. It is a fundamental right that must be ensured without distinction of race, religion, or social condition.”

Right after reading, the patients were asked “Can you speak comfortably?”, and answers could be “yes” (TT+); “uncertain” (TT±); or “no” (TT−). The TT− was considered a criterion to interrupt the TT. Other criteria for interrupting the test were similar to the CPET and 6MWT.

### TT protocol based on the 6MWT

A continuous incremental protocol was conducted with speed or incline increments at the beginning of each stage. An equation was used to calculate the predicted 6MWT distance (6MWDpred).^20^


$$\begin{array}{cc} 6MWDpred & =890.46-(6.11\times \text{ age })+\left(0.0345\times {age}^{2}\right)\\ & +(48.87\times \text{ sex })-(4.87\times BMI) \end{array}$$

Knowing the 6MWDpred, mean speed was estimated as follows:  TTspeed (km/h)=(6MWDpred[m]×10[min])/1000 ; equivalent to 100% of estimated mean speed during the 6MWT. We also calculated the percentages corresponding to each TT stage, initiating at 70% and increasing 10 percentage points every 2 minutes until 110% of TTspeed. The treadmill incline was maintained at 2% until the first stage at 110% of TTspeed. After that, 2 percentage points were increased each stage until the end of the protocol (Figure 1S, Supplementary Material).

### Statistical analysis

Data were analyzed using the Statistical Package for the Social Sciences software, version 20.0 (SPSS, IBM Corporation, Armonk, USA). Continuous variables were described as mean and standard deviation or median and interquartile range, according to data normality. The normality of the data distribution was tested with the Shapiro-Wilk test.

Variables with normal distribution were compared using the repeated measures ANOVA, followed by Bonferroni’s post-hoc, while the Friedman’s tests was used for variables with non-normal distribution. Pearson’s or Spearman’s correlation coefficient assessed association between variables. The significance level adopted in the statistical analysis was 5%.

### Sample size calculation

The sample size was calculated using GPower 3.1 in a pilot study with 5 participants, considering bidirectional significance level (α) of 5% and power of 80% (β = 0.20). Based on the correlation between HR during TT stages (TT+ and TT±) and VT1 (TT+: r = 0.95; TT±: r = 0.96) and HRpeak at 6MWT (TT+: r = 0.56; TT±: r = 0.57), the minimal estimated sample size was 20 patients.

## Results

We evaluated 30 patients, and 8 of them were excluded due to musculoskeletal alterations (n = 3), measurement error (n = 3), angina (n = 1), and periumbilical pain (n = 1). Of the 22 included patients, 13 were males (59.1%) (Table 1).

**Table 1:** – Descriptive characteristics of subjects

Variable	Mean (SD)	n	%
**Age (years)**	61.1 (8.5)		
**Body mass index (kg/m^2^)**	29.5 (4.1)		
**HR (bpm)**			
Rest (before CPET)	73.6 (1.8)		
Rest (before 6MWT)	74.4 (2.2)		
Rest (before TT)	74.1 (1.9)		
VT1	100.9 (14.8)		
VT2	119.0 (16.1)		
6MWT	119.4 (20.1)		
TT+	101.1 (14.7)		
TT±	105.4 (15.7)		
TT−	121.4 (18.4)		
**Predicted distance in 6MWT (m)**	530.7 (42.9)		
**Real distance in 6MWT (m)**	577.8 (87.9)		
**Real distance in relation to predicted in the 6MWT (%)**	108.9 (14.9)		
**Cardiac diagnosis**			
Coronary artery disease		19	86.4
Heart failure		1	4.5
Coronary artery disease + heart failure		9	40.9
Hypertrophic cardiomyopathy		1	4.5
Heart valve diseases		2	9.1
**Comorbidities**			
Obesity		9	40.9
Dyslipidemia		15	68.2
Hypertension		19	86.4
Diabetes mellitus		9	40.9
Ex-smoker		9	40.9
**Angioplasty**		13	59.1
**Cardiac surgery**			
Myocardial revascularization		10	45.5
Valve replacement		2	9.1
**Time in the rehabilitation program (months)**			
1–6		11	50.0
6–12		1	4.5
12–24		4	18.2
≥24		6	27.3

CPET: cardiopulmonary exercise test; HR: heart rate; 6MWT: 6-minute walk test; TT: talk test; TT+: last positive stage of TT; TT±: first stage of TT equivocal; VT1: first ventilatory threshold; VT2: second ventilatory threshold. Source: elaborated by the authors.

As shown in Table 1, no significant difference was found between HR at rest before the CPET, TT, and 6MWT (p = 1.0).

Figure 1 presents the comparison between HR at VTs, TT+, TT±, TT−, and HRpeak in the 6MWT. We found a significant difference between HRpeak in the 6MWT and HR at VT1, TT+, and TT± (p < 0.001, p = 0.001, and p = 0.005, respectively). No difference was observed between HR at VT1 and TT+ (p = 0.987) and TT± (p = 0.154) (Figure 1A) and between VT2 and TT− (p = 0.383). HRpeak in the 6MWT was similar to HR at TT− (p = 0.68) and VT2 (p = 0.92) (Figure 1B).

**Figure 1:** – A: Comparison of HR between VT1, stages TT+ and TT±, and 6MWT. B: Comparison of HR between VT2, TT– and 6MWT. Source: elaborated by the authors. Note: Due to non-normal and normal distribution, Friedman’s test was used for Figure 1A variables, and repeated measures ANOVA was used for Figure 1B variables, respectively.

A moderate correlation was found between TT+ and VT1. Nonetheless, HR at TT± was not correlated with HR at VT, and HRpeak in the 6MWT. HR at TT− was strongly correlated with VT2. HRpeak in the 6MWT did not correlate with HR at VT1 but correlated with HR at VT2 and TT−. Table 2 presents all the correlations of HR at TT stages, VT, and HRpeak in the 6MWT.

**Table 2:** – Correlations of HR at TT stages, VT1, and HRpeak in the 6MWT

	HR at VT1		HRpeak (6MWT)	

	rho	p	r	p
TT+	0.479	0.024*	0.384	0.078
TT±	0.412	0.057	0.357	0.103

HR: heart rate; HRpeak: peak heart rate; 6MWT: six-minute walk test; TT+: last positive stage of TT; TT±: first stage of TT equivocal; VT1: first ventilatory threshold. *p < 0.05. Source: elaborated by the authors.

## Discussion

This is the first study comparing the HR assessed at VTs of the CPET with the HR at stages of an independent and individualized TT protocol, and during a field test (6MWT). HR at TT+ and TT± were similar to HR at VT1, while HR at TT− and HRpeak in the 6MWT were similar to HR at VT2. HR at TT+ and TT− correlated with HR at VT1 and VT2, respectively. No correlation was found between HRpeak in the 6MWT and HR at TT stages.

The most recognized individualized AE prescription methods are the maximal oxygen uptake and VTs identified using the CPET.^1,4^ If a CPET is unavailable, graded exercise test may be used to obtain maximal cardiovascular parameters. In the absence of these tests, a functional assessment and AE prescription are usually carried out based on a field test, such as 6MWT and TT.^8^ Although the TT stages are physiologically related to the VTs, their use for AE prescription is still scarce, mainly for cardiac patients.

In the present study, we explored a TT protocol and found correlations between HR at VT1 and TT+ and TT±. The TT does not have a standardized protocol and may be performed with different equipment, load progressions, and stage lengths, and the speech can be challenged using paragraphs or counting.^16^ Despite using different methods, studies have demonstrated similarities between HR and oxygen uptake at TT stages and VTs in patients with cardiac disease, suggesting that TT stages may be used to prescribe AE.^14,15,21,22^

Our results were similar to Brawer et al. (2006), who applied the TT in patients with stable coronary artery disease and demonstrated no difference in estimating VT1 and the viability of prescribing AE using HR according to TT.^22^ In addition, a strong correlation was found between HR at TT− and VT2. Although there is a lack of studies comparing HR between these two moments in patients with cardiovascular disease, associations between oxygen uptake and HR at TT− and VT2 were found in healthy individuals and athletes,^23,24^ therefore suggesting that TT− may guide AE prescription and reflect the prescription based on VT2.

Another method commonly used at cardiovascular rehabilitation programs for prescribing AE intensity is based on the 6MWT. Gremeaux et al. (2011) compared the effects of 3 individualized exercise training prescriptions in individuals training at a moderate intensity. They found similar values between HRpeak in the 6MWT and the recommended target HR. Another study by Calegari et al. (2021) compared HR at VT1 during the last 30 seconds of the 6MWT in patients with coronary artery disease on β-blockers treatment. The authors found an agreement between HR assessed at the end of the 6MWT and VT1, suggesting that HR at the end of the 6MWT was adequate to prescribe and monitor AE in this population.^25^

These findings support the idea of using the HR at 6MWT as an exercise intensity prescription method, with HR measured in the 6MWT being similar to HR assessed at VT1. However, we found differences between HRpeak in the 6MWT and HR at VT1 in our study, while HRpeak in the 6MWT was similar to HR at VT2 and at TT−. According to the most recent recommendations, AE prescription should be performed between the first and second VTs;^1^ our data suggest that HRpeak in the 6MWT could not be adequate to prescribe lower limit AE intensity in patients with chronic cardiovascular disease. Furthermore, the HRpeak at 6MWT, such as VT2 and TT−, may represent the upper limit of AE prescription.

This similarity has never been described in the literature, and some aspects of the present study may have influenced this outcome. Patients were part of a cardiovascular rehabilitation program that regularly performs the 6MWT. In addition, regarding the walked distance, our sample reached a distance walked above 100% of the predicted, demonstrating little impairment of functional capacity. Moreover, the 6MWT was performed in an open field, whereas CPET and TT were performed in controlled environments. In addition, previous studies used HR from the final minute of the 6MWT instead of HRpeak during the test, which was used in our analyses.

Although commonly used in cardiovascular rehabilitation, the 6MWT requires physical space because of the 30-meter-long track. Furthermore, the test is self-paced and presents inter- and intra-individual variations of speed and performance.^18^ On the other hand, the TT requires less physical space, may be performed in a cycle ergometer or treadmill, follows an incremental ramp protocol similar to CPET, and is easy to apply and equivalent to the reference standard.

This study is not free of limitations. Patients were part of a phase III cardiovascular rehabilitation program and possibly presented better physical conditions than patients in the earlier phases of rehabilitation. Moreover, the perception of comfort speech is subjective, and data were lost due to measurement error.

Comparisons between TT and CPET were similar to the literature, except between TT and 6MWT. We believe that TT presents parameters associated with physiological and CPET variables (e.g., VTs). Our results also showed the clinical applicability of TT as an easy, safe, and individualized tool for AE prescription.

## Conclusion

According to the proposed protocol, HR at TT+ and TT− reflect HR at VT1 and VT2, respectively, demonstrating that TT is an objective and low-cost test to assist AE prescription in patients with chronic cardiovascular conditions. In contrast, AE prescription using HRpeak in the 6MWT must be carried out with caution since some studies have shown its correlation with VT1, differently from what was found in our study, in which HRpeak in the 6MWT was similar to HR at VT2 and TT−. We suggest further randomized clinical trials using TT parameters to prescribe AE in patients with cardiovascular diseases.
